# State Control of Consumption

**Published:** 1900-03

**Authors:** 


### STATE MEDICINE.

### STATE CONTROL OF CONSUMPTION.

### REPORT OF THE COMMITTEE ON HYGIENE OF THE MEDICAL SOCIETY OF THE STATE OF NEW YORK.[1]

YOUR committee on hygiene would respectfully report:

Directed by our sense of responsibility, by the logic of the situation, as well as by the resolution of the society of last year, your committee has given particular consideration to the subject of state sanatoria for the cure and prevention of consumption.

We think it expedient to trace the growing interest which this society has taken in the subject of consumption, since the discovery of its infectious nature. The quickening influence of Koch's immortal discovery appears in this society's transactions for 1891, the scientific event of the meeting of that year being a Discussion on tuberculosis, including the following communications: Its history, by H. R. Hopkins; Its pathology and etiology, by Heneage Gibbs; tuberculous manifestations in the upper air tract and special treatment, by John O. Roe; Treatment, including prophylaxis as related to climate, by Samuel B. Ward; Treatment as related to therapeutics, by E. L. Shurly; Inoculations with Professor Koch's tuberculin, by A. Jacobi; On Koch's treatment in Mount Sinai and New York Polyclinic Hospitals, by H. N. Heineman.

In 1892, The danger of Milk from tuberculous cows, by Edward F. Brush.

In 1896, Shall the state undertake to restrict the spread of tuberculosis, by Jno. L. Heffron. This paper concludes as follows:

(1) Tuberculosis is an infectious and curable disease, capable of restriction; (2) that the state should compel the registration of every case of tubercular disease; (3) that circulars of information as to the nature, communicability and sanitary cure of tubercular disease should be sent to those afflicted with the disease, and to those attending them; (4) that instruction as to the nature of contagious and infectious diseases, and the practical methods of their control, should be given to all senior pupils in public grammar schools; (5) that all owners and trustees of places of public entertainment, including churches and schools, and all public carriers, should be required to prevent contamination of their halls and conveyances and to disinfect them; (6) that the hopelessly ignorant, wilfully careless and vicious, afflicted with tuberculosis should be isolated in special hospitals provided by the state.

---

1. Presented at the 94th Annual Meeting at Albany, January 30, 1900.

In 1897, The necessity of new methods of early diagnosis in tubercular disease to curative treatment, with remarks on treatment, by J. B. Ransom.

In 1898, What shall the state and county do for the consumptive? by John H. Pryor; The hygienic management of dairies, by E. F. Brush; The municipal control of milk supply in cities and villages, by W. H. Heath.

In 1899, The detection of tubercular infection in second-hand clothing, by William G. Bissell; Relations of the state to the consumptive from the standpoint of political economy, by George W. Brush; The relation of the state to the consumptive, with special reference to prevention and state care of the incipient consumptive by John H. Pryor.

The steadily developing interest in this important matter takes definite shape and precise and formal expression this year (1899) in the following resolutions, offered by Dr. George W. Brush, and unanimously adopted :

WHEREAS, It has been shown by the facts presented to this Society, and substantiated by statistics, that tuberculosis caused the death of more than thirteen thousand of the population of this State; and

WHEREAS, The researches of Science, and the results of experience, prove this disease to be a communicable one, and one easily preventable by proper knowledge on the part of the people; and

WHEREAS, There are no hospitals for the proper treatment of this disease, either State or Municipal; therefore,

*Resolved*, That the Medical Society of the State of New York urges upon the Legislature of New York State speedy legislation for the establishment of Sanatoria, where those afflicted with this disease may be treated, and of Sanitaria in the suburbs of large cities; and

*Resolved*, That the Committee on Legislation of this Society be directed to urge the passage of some measure by the present Legislature looking toward the establishment of a State Sanatorium.

*Resolved*, That these resolutions be printed, and a copy sent to the Governor, and to each member of the Legislature now in session.

At the same session the society adopted a resolution offered by Dr. Pryor, That the subject of state control for the prevention of tuberculosis be referred to the committee on hygiene.

Your committee on hygiene has the conviction that the principles involved in the question of the prevention of tuberculosis by the power and authority of the state are so plain, simple and recognised by all intelligent persons as to require neither argument nor discussion but only brief statement. The fundamental proposition is, that all states have the right, and that all efficient states have the power to protect the lives and the property of the people. The enormous loss of life and destruction of property from tuberculosis, human, bovine and animal, is the energising fact which should call into action, give purpose, direction and test of this ability of the state to show its sovereignty.

*Salus populi suprema lex.*

In advancing from general considerations to those of greater particularity, in supporting the recommendation of the society for the establishment of state sanatoria for the treatment of cases of incipient consumption, we have the hopeful and comforting assurance that the practice is not visionary or experimental, but is sound and rational, and supported by the best judgment of the men of the widest experience, upon evidence which is absolutely demonstrative.

And further, that this practice has the additional claim to professional confidence, in that it rests upon the well known principles, early recognition, isolation and disinfection--that invincible triad, which in the warfare against the communicable diseases has won those glorious victories, the pride and boast of preventive medicine, the brightest page in all medical history.

And yet, still further, that the treatment used in these sanatoria, may calmly and confidently claim the attention of a sovereign state because of its ancient and honorable lineage, having come to us from Hippocrates, Aretëus and Galen, with approbation of the great minds of medicine of each century and of all countries, and because of its simplicity, naturalness and efficiency.

Sanatoria for the treatment of consumptives are today in successful operation in almost every civilised country of the world—Austria, Italy and Poland each has one; Hungary, Belgium, Scotland, Ireland and Holland, each two; Denmark, four; Norway, five; Switzerland, eight; Russia, eleven; France, twenty-one; England, twenty-two, and Germany, forty-three.

The United States has thirty-three distributed as follows: Alabama, two; Colorado, three; Illinois, three; Maryland, one; Massachusetts, four; New Mexico, four; New York, ten; North Carolina, two; Pennsylvania, four, and in Ontario there are two. Those in this country are all private institutions, excepting one state sanatorium in Massachusetts.

Sanatoria for consumptives are not an experiment; the hold which these institutions have upon professional and popular confidence, is the result of critical observation upon their work for many years, more than a third of a century; not in one locality alone where singular environment not to be repeated elsewhere may have been the factor in treatment, but in many localities with wide variations as to altitude, humidity, temperature, sunshine and winds; not upon cases of consumption from a single race alone, but upon consumptives of many races; not upon patients coming from a selected social class, but rather upon cases from all classes--high and low, rich and poor, vicious and worthy, peasant, serf and nobility.

Again, it is to be noted that the growth of these sanatoria has been slow. Brehmer opened his in 1859, from small beginnings; by reason of successful work they have grown in proportions and increased in numbers; planted by the courage and faith of individuals, they have won the confidence of the medical profession, the philanthropist, the community and at length that of the government of the state.

Medical opinion is notedly conservative; the demonstration of the infectiousness, the communicability, the preventability of consumption was made nearly twenty years ago, nearly the span of a generation has passed since that revolutionising fact, and the revolution still tarries.

The watchman from the lookout reports that signal fires are burning here and there and there upon various mountain peaks; but in the valleys and plains the people still sleep—and year by year the tolling bells continue to number the enormous death-rate from consumption.

The discoveries of Koch and his supporting followers, is a trumpet call to action in the most beneficent and inspiring enterprise that ever engaged the energy, wisdom and enthusiasm of man—the abolition of the plague, the pestilence of human consumption.

To whom does this call come? To every man whose mind and opportunity has permitted him intelligently to study the literature of medicine upon this subject, and whose character is such as to enable him to engage in forceful action.

And who are they who will be found in opposition? All men who, refusing to accept the demonstrative labors of clinicians, bacteriologists and pathologists, hold that consumption is chiefly communicated by hereditary transmission, capable of stepping over one or more generations; all men whose love of ease is structural, whose desire to be known as fair-minded keeps them from having effective convictions, when convictions demand action; who hold it safer to stop at opinions, because opinions may be so balanced as not to disturb conscience or interrupt repose; all impracticable men, who decline to consider or to discuss plans for the foundations of the house, the nature and proportions of its walls, until these peculiar ideas are accepted as to the color scheme for tinting the ceiling of the third story bedrooms; finally, all over-bred, thin blooded and burned-out men, whose courage, faith and effectiveness fully but fitly expresses itself in the pessimistic protest, "Am I my brother's keeper?"

In conclusion, your committee on hygiene emphatically and unreservedly commends the subject of state prevention of consumption, as the highest and most sacred privilege and obligation of the state.

That our increasing ability by means of bacteriological aid to make more accurate and early diagnosis of consumption, makes the already proven efficiency of treatment in sanatoria hopeful beyond expectation, and worthy the confidence and liberal support of the state.

That nature has, in a most generous measure, endowed the Empire State with those fortuitous conditions, virgin forests on foot-hills and mountain sides, streams of pure water, extended, uplifted and inspiring scenery, varieties of altitude with exposure to sun and with shelter from winds—all the provision of nature for nature's beneficent work. This constitutes an occasion, an opportunity, a responsibility which may no longer be disregarded; this demands that the Medical Society of the State of New York shall "speak to the people that they go forward."

We advise and recommend that this society cease not from its efforts until the State of New York affords to its incipient consumptives, the beneficent and hopeful treatment of state sanatoria.

(Signed) HENRY R. HOPKINS,
J. H. MOSHER,
M. A. VEEDER,
H. L. ELSNER,
GEORGE SEYMOUR.

---

TOOTHACHE.

| | | | |
|---|---|---|---|
| ℞ Extract of opium | } āā | 1 | (gr. xv.) |
| Powdered camphor | | | |
| Balsam Peru | | | |
| Mastic | | 2 | (gr. xxx.) |
| Chloroform | | 20 | (ʒv.) |

Wet a small piece of absorbent cotton with this solution and insert in cavity of tooth.—*L'Union Médicale.*

